# Pediatric Adrenocortical Tumors: What They Can Tell Us on Adrenal Development and Comparison with Adult Adrenal Tumors

**DOI:** 10.3389/fendo.2015.00023

**Published:** 2015-02-18

**Authors:** Enzo Lalli, Bonald C. Figueiredo

**Affiliations:** ^1^Institut de Pharmacologie Moléculaire et Cellulaire CNRS, Valbonne, France; ^2^University of Nice-Sophia-Antipolis, Valbonne, France; ^3^Associated International Laboratory (LIA) NEOGENEX, CNRS, Valbonne, France; ^4^Federal University of Paraná, Curitiba, Brazil; ^5^Instituto de Pesquisa Pelé Pequeno Principe, Curitiba, Brazil

**Keywords:** cancer, adrenal glands, adrenal gland neoplasms, adrenal cortex, genetic

## Abstract

Adrenocortical tumors (ACT) in children are very rare and are most frequently diagnosed in the context of the Li-Fraumeni syndrome, a multiple cancer syndrome linked to germline mutations of the tumor suppressor gene *TP53* with loss of heterozygosity in the tumors. A peak of children ACT incidence is present in the states of southern Brazil, where they are linked to the high prevalence in the population of a specific *TP53* mutation (R337H). Children ACT have specific features distinguishing them from adult tumors in their pathogenetic mechanisms, genomic profiles, and prognosis. Epidemiological and molecular evidence suggests that in most cases they are derived from the fetal adrenal.

## Dynamics of Human Adrenocortical Morphology and Hormone Secretion during Development and Postnatal Life

The adrenal gland is a continuously evolving endocrine organ from the developmental to the elder age. In humans, adrenal gland development begins at 3–4 weeks of gestation by a condensation of the coelomic epithelium lining the abdominal cavity, followed at 4–6 weeks of gestation by proliferation and migration of coelomic epithelial cells, and subsequent differentiation of fetal adrenal cortical cells into two distinct zones (inner fetal zone and outer definitive zone) at 8–10 weeks of gestation, while neural crest-derived cells start to infiltrate the gland at 7–8 weeks of gestation to give origin to adrenomedullary cells (1). Starting from around the ninth week of gestation, the embryonal adrenal is surrounded by the adrenal capsule formed by mesenchymal cells. Fetal adrenal cells, which are large and rich in lipids, express the steroidogenic enzyme CYP17, which enables them to produce high levels of DHEA and its sulfoconjugate DHEAS, which play a key role for the maintenance of pregnancy, being metabolized into estrogens by the placenta (1, 2). By the end of the second trimester of gestation, a distinct zone (transitional zone) differentiates between the definitive and fetal zones, which express *HSD3B2*, this way starting cortisol synthesis in the fetus. Close to birth, *HSD3B2* is expressed in the definitive zone, which acquires the capacity to synthesize the mineralocorticoid hormone aldosterone. Cell proliferation in the fetal adrenal is mainly localized in the outer definitive zone, followed by centripetal migration and differentiation into fetal zone cells, which subsequently die from apoptosis in the center of the gland. This streaming process of adrenocortical cell differentiation continues during the whole life, as shown by studies in the mouse (3–5).

Starting shortly after birth, a rapid, dramatic remodeling of adrenal cortex structure takes place, with massive shrinkage of the gland due to apoptosis of the fetal zone and progressive differentiation of the *glomerulosa*, *fasciculata*, and *reticularis* zona, which are the hallmark of the adult adrenal (1). Defects in this process may cause the cytomegalic form of adrenal hypoplasia congenita, a syndrome of adrenal insufficiency due to altered postnatal adrenocortical differentiation due to mutations in the *NR0B1* (*DAX-1*) gene [reviewed in Ref. (6)]. Studies in pre-term neonates have shown that parturition itself is the cause for fetal adrenal involution (7), suggesting a crosstalk between placenta and fetal adrenal in reciprocal maintenance. Remarkably, postnatal adrenal remodeling also takes place in the mouse adrenal cortex, where an inner zone adjacent to the medulla termed zone X, that lineage tracing experiments have shown to be derived from the fetal adrenal (8), regresses after puberty in males and after the first pregnancy in females.

After being suppressed following the regression of the fetal zone, adrenal production of DHEA/DHEAS starts to progressively increase again by around 8 years of age. This phenomenon is termed adrenarche and is concomitant with full differentiation of the *reticularis* zone, which expresses *CYP17* but not *HSD3B2*. Moreover, in the *zona reticularis*, CYP17 has an increased ratio of 17,20-lyase to 17α hydroxylase activity (which favors DHEA production) compared to the *zona fasciculata*, probably due to increased serine phosphorylation and increased abundance of cytochrome b5 (CYB5), which allosterically stimulates 17,20-lyase activity of CYP17 (9). DHEA/DHEAS levels continue to increase until adulthood and then progressively decline (adrenopause) reaching pre-adrenarche levels by the ninth decade, correlating with progressive atrophy of the *zona reticularis* (10).

## Adrenocortical Tumors in Children and Adults: Similarities and Differences

Adrenocortical tumors (ACT) are among the most common neoplasms in humans and are frequently detected by hazard during diagnostic procedures for other medical issues (incidentalomas), in the great majority of cases remaining clinically silent and having a completely benign prognosis. In contrast, adrenocortical malignancies (adrenocortical carcinomas or ACC) are very rare, with a general incidence of 0.7–2 cases/million/year, with a maximum between 40 and 50 years of age and a higher frequency in women than in men (11). They become clinically evident with signs and symptoms due to hormone excess (Cushing’s syndrome, androgen excess) and/or local symptoms (pain, abdominal discomfort). The prognosis of ACC is still poor, with an average 5-year overall survival around 40%, which is influenced to a great extent by tumor stage at diagnosis. Some histopathological parameters (Weiss score ≥3, Ki-67 index >10%) also have negative prognostic value (11).

Adrenocortical tumors in children under 15 years of age are even rarer. Their worldwide incidence has been estimated at 0.3/million/year with a bimodal peak under the age of 5 and after 10 years and they also affect girls more frequently than boys. The main reason why ACT in children become clinically evident is virilization, which may be associated to Cushing’s syndrome. Overall survival at 5 years after diagnosis in children with ACT is better than in adult patients, approximating 50%. Favorable prognostic factors are younger age (<4 years), stage I at diagnosis, tumor weight ≤200 g, volume <200 cm^3^, and presence of virilization alone (12). It is noteworthy that in children ACT, the Weiss score is not a reliable system to assess malignancy (13–15) (Table 1).

**Table 1:** **Distinctive features and common characteristics of ACT in children and adults**.

	Children ACT	Adult ACC	References
Peak age at diagnosis	3–4 years	40–50 years; peak extending into the seventh decade	(10, 11, 26)
Clinical presentation	Most often virilization; may be associated with Cushing’s syndrome	Cushing’s syndrome or hypertension; may be associated with virilization	(10, 11)
Prevalence	Worldwide: 0.3 cases/million/year; southern Brazil: 3.4–4.2 cases/million/year	0.7–2/million/year for ACC	(10, 11)
Most common genomic alterations	11p15 LOH; 9q34 gain; 4q34 loss	Complex pattern	(46, 70–74, 86–92)
Genetic syndromes
Overall LFS	>50%	Sporadic germline *TP53* mutations	(26, 29, 30)
Endemic germline *TP53* R337H (Brazil)	>93%	<20%	(10)
Beckwith-Wiedemann syndrome	Yes	Uncommon	(17, 47)
FAP	Uncommon	Yes	(47, 48)
MEN1	Uncommon	Yes	(47, 49)
Lynch syndrome	Uncommon	Yes	(47, 50)
NF1	Uncommon	Yes	(47, 51)
Prognostic relevance of
Pathological (Weiss) score	Low	High	(13–15)
Ki-67 index	Unknown	High	(11)
Prognostic relevance of
*TP53* mutations	No (germline)	Yes (somatic)	(26, 29, 30)
IGF2 overexpression	No	Yes	(18–22)
NOV down-regulation	No	Yes	(19, 52)
SF-1 overexpression	No	Yes	(31, 78, 81, 82)
HLA class II down-regulation	Possible	No	(19, 22)
*DLGAP5-PINK1* expression	No	Yes	(54, 56)
*BUB1B-PINK1* expression	No	Yes	(54, 56)
Molecular pathways involved
IGF2	Yes	Yes	(18–22)
p53/Rb	Yes (*TP53* mutations)	Yes (*TP53/CDKN2A/RB1* mutations)	(26, 57, 66, 85)
Beta-catenin	Yes (*CTNNB1* mutations)	Yes (*CTNNB1/ZNRF3* mutations)	(57, 66, 85)
Chromatin remodeling	Yes (*ATRX* mutations)	Yes (*MEN1/DAXX/ATRX/MED12/TERT* mutations)	(66, 85)

Childhood malignancies have long been associated to congenital defects (16), which suggest that they may be considered as a degeneration of normal developmental processes. Children ACT are a typical example since they can be found in the context of two genetically determined syndromes, Beckwith-Wiedemann and Li-Fraumeni.

**Figure 1:**
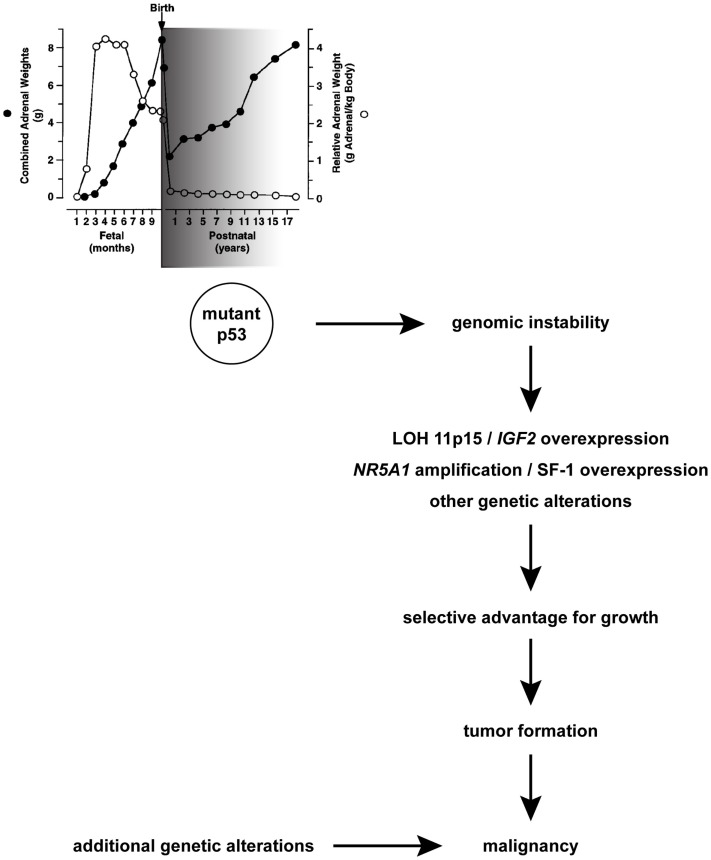
**A hypothesis for *TP53* role during physiological adrenocortical remodeling in early postnatal life and pathogenesis of children ACT**. A window of sensitivity (with an early peak; shaded in grey) of human adrenal to defective p53 function exists during the first years after birth when its physiological involution takes place. Mutant p53 may favor genomic instability, which in some cells may cause LOH of 11p15 and gain/amplification of *NR5A1*, which confer those cells a selective advantage for growth and lead to tumor formation. Additional genetic alterations arising in this mutation-prone background may cause malignancy. Combined adrenal weight is indicated with black circles. Relative adrenal weight in relationship to body weight is indicated with white circles. Adapted in part from Ref. (1) with permission from Endocrine Society Press.

(1) Adrenocortical hyperplasia and neoplasms of variable malignancy are common in Beckwith-Wiedemann syndrome, a systemic overgrowth syndrome caused by genetic defects as uniparental disomy in the 11p15 chromosomal region (17), which cause overexpression of the IGF2 growth factor in the great majority of cases. Loss of heterozygosity (LOH) of the 11p15 region is a systematic finding, not related to prognosis (18–20), in children ACT, leading to *IGF2* overexpression from the paternal allele. Similarly, *IGF2* is expressed at high levels in the fetal adrenal where it has an important role to regulate proliferation and steroid production (1). Conversely, *IGF2* overexpression and abnormalities in the 11p15 region are a marker of malignancy in ACT of adults (21, 22). In mouse models, *Igf2* overexpression in the adrenal induces tissue hyperplasia but is not able to induce malignant tumorigenesis *per se* (23, 24).
(2) Adrenocortical tumors are a distinctive feature of Li-Fraumeni syndrome (LFS), a multiple cancer syndrome due to germline mutations in the *TP53* tumor suppressor gene [(25); reviewed in Ref. (26)] encoding p53, a transcription factor that has a pivotal role in preserving genome integrity and activating apoptosis of cells bearing irreparable DNA damage (27). It has been shown that in LFS, excessive DNA copy number variation exists in the patients’ germline, which may predispose to cancer (28). Due to its rarity and its characteristic association with LFS, discovery of an ACT in a child is an absolute indication for researching *TP53* mutations in the proband and in his/her parents as well indicative for genetic counseling. Conversely, germline *TP53* mutations are much less common in adults with ACC (29, 30) (Table 1). The high incidence of children ACT in LFS suggests that normal p53 function is required for the physiological process of postnatal fetal adrenal regression (Figure 1). In the absence of p53, genetic alterations may accumulate in the adrenal driving proliferation [such as *NR5A1* overexpression, (31, 32); see below section on Whole Genome Studies in Children and Adult ACT Reveal Important Drivers for Tumorigenesis and LOH of 11p15 leading to *IGF2* overexpression (18–20)] of specific cellular clones. This increased proliferative capacity may favor the emergence of further genetic alterations ultimately leading to clonal expansion and tumorigenesis [reviewed in Ref. (33)].

In classical LFS, due to *TP53* mutations that completely abolish protein function, the lifetime incidence of cancer in carriers is close to 100%. However, low-penetrance mutated *TP53* alleles exist that can increase the risk of developing cancer only in a fraction of carriers (34). A remarkable example of that situation exists in southern Brazil. In that geographical region, children ACT prevalence is at least 15-fold higher than in the rest of the world (10). This is related to a specific germline *TP53* mutation (R337H) (35, 36), whose prevalence is very high (0.3%) in the population but whose penetrance to produce ACT in children has been estimated at only about 2% (37). However, the *TP53* R337H mutation has also been reported to be associated to other cancers in the Li-Fraumeni spectrum (38–41) and so its overall penetrance is still unknown. R337 is a conserved arginine residue in the C-terminal tetramerization domain of p53 whose mutation to histidine destabilizes p53 tetramer formation in conditions of elevated temperature and pH (42). It has been shown that a founder effect is responsible for the spreading of the *TP53* R337H mutation in the population of southern Brazil (43, 44). An about 0.5 Mb identical by descent haplotype in 17p13 encompassing the *TP53* gene carrying the R337H mutation is conserved in all carriers of the mutation (45, 46). A newborn screening and surveillance program of the *TP53* R337H mutation carriers in the state of Paraná has proven to be successful to detect ACT in children at an early stage and to treat it with better therapeutic results compared to children who did not undergo surveillance (37).

Apart from rare cases of germline *TP53* mutations, as mentioned before (29, 30), ACC in adults may also be associated to other hereditary conditions in some uncommon cases [reviewed in Ref. (47)]: familial adenomatous polyposis (FAP) (48), multiple endocrine neoplasia type1 (MEN1) (49), Lynch syndrome (50), and neurofibromatosis type 1 (NF1) (51) (Table 1).

## Genome-Wide Studies in Children and Adult ACT

### Distinct patterns of coding genes expression in children vs. adult ACT

Children ACT can be readily differentiated from age-matched normal adrenals by unsupervised clustering based on their gene expression profiles (19). As reported before, *IGF2* is the single gene that is most highly up-regulated in children ACT, while genes in the 11p15 region expressed from the maternal allele (*KCNQ1*, *CDKN1C*) are among the most strongly down-regulated transcripts. These data are consistent with the systematic LOH of 11p15 in those tumors, with conservation of the paternal allele and loss of the maternal allele (18, 20). Genes belonging to growth factor receptor and mitogen-activated kinase pathways are also dysregulated in children ACT. This suggests that those signaling pathways may be targets for therapeutic intervention. Furthermore, *HSD3B2*, a steroidogenic enzyme involved in the synthesis of aldosterone and cortisol and expressed in the *glomerulosa* and *reticularis* zones of the adult adrenal cortex and its transcriptional regulators *NR4A1* and *NR4A2* are strongly down-regulated in children ACT, lending further support to the hypothesis of their derivation from the fetal adrenal. This is also suggested by the finding that global gene expression profiles of children ACT are significantly correlated with those present in the fetal adrenal. Another strongly down-regulated gene in children ACT is *NOV*, encoding a secreted multimodular protein that has a pro-apoptotic function on adrenocortical cancer cells (52). In the study by West et al. (19), a set of 52 differentially expressed genes between adrenocortical adenomas and carcinomas (as distinguished by histological parameters) was identified. It is noteworthy that those included some transcripts encoding HLA class II molecules. Down-regulation of class II expression may represent a mechanism to escape immune surveillance, which could contribute to malignancy. Since malignancy markers are dramatically lacking for children ACT, it will be very important to confirm those data in larger series of patients. However, a recent immunohistochemical study failed to detect consistent HLA class II immunoreactivity in children ACT, both benign and malignant (53).

Unsupervised clustering of gene expression profiles of adult ACT allowed to distinguish two groups, termed C1 and C2 in the study by de Reyniès et al. (54). The C1 group could be further subdivided into C1A and C1B, which correspond to unfavorable and favorable outcome, respectively. Those results were confirmed by another independent study (55), which also confirmed that *IGF2* overexpression is associated to malignancy in adult ACC. From gene expression data, de Reyniès and collaborators identified both a signature for malignancy based on the analysis of the expression of two genes (*DLGAP5/PINK1*) and a two-gene (*BUB1B/PINK1*) molecular predictor of overall survival for patients with ACC (54). Remarkably, those molecular markers were confirmed to be valid prognostic indicators in adult but not in children ACT in a study on patients from southern Brazil (56) (Table 1). Further studies showed that tumors classified in the C1A group could be further divided into two subgroups each one bearing either *TP53* or *CTNNB1* (beta-catenin) mutations and in a third subgroup with no other known mutation (57). The importance of the activation of the beta-catenin pathway for adrenocortical tumorigenesis is also shown by studies in mouse models [(23, 24, 58); reviewed in Ref. (59)].

### microRNA sets differentially expressed in children and adult ACT

In the only study published to date investigating miRNA expression profiles in children ACT, a distinct subgroup of miRNA was found to be differentially expressed in tumor samples compared to age-matched normal adrenal cortex (60). This subgroup included *miR-99a* and *miR-100*, which are down-regulated in children ACT and are able to down-regulate expression of IGF-1R (the receptor for IGF2), mTOR, and its associated protein raptor in adrenocortical cell lines. These proteins are up-regulated in children ACT and their pharmacological blockade is able to significantly decrease adrenocortical cancer cell proliferation (60–63). These results show that *miR-99a* and *miR-100* have an important role in children ACT by the modulation of growth factor signaling through the IGF-1R–mTOR pathway.

On the other hand, several studies reported data on miRNA expression profiles in adult ACT. Those studies show in general only limited overlap [reviewed in Ref. (64)]. Nevertheless, most studies detected overexpression in ACC of *miR-483-5p* and/or *-3p*, whose gene is situated in an intron of *IGF2* and may have an independent oncogenic function (65). *miR-483-3p* was also found up-regulated in children ACT in the study by Doghman et al. (60). Other miRNAs that display similar differential regulation in children and adult ACT are *miR-503* (up-regulated), *miR-195*, *miR-214*, and *miR-375* (down-regulated). A recently published integrative analysis of genomic alterations in adult ACC (66) found up-regulation of miRNAs belonging to the *miR-506-514* cluster on chromosome Xq27 and down-regulation of the expression of the *DLK1-MEG3* miRNA cluster on chromosome 14q in one subgroup of samples with favorable prognosis (C1B; see below section on Whole Genome Studies in Children and Adult ACT Reveal Important Drivers for Tumorigenesis). There is of great interest for the potential use of circulating miRNAs as biomarkers of malignancy in ACC (67–69).

### Whole genome studies in children and adult ACT reveal important drivers for tumorigenesis

The first studies analyzing children ACT genome copy number alterations by comparative genomic hybridization (CGH) reported patterns of recurrent gains and losses (70–72). In particular, one of the most common alterations found in almost all cases of children ACT investigated was the gain/amplification of 9q34. Gains in this region were also reported in some studies of chromosomal alterations in adult ACT (73, 74). In close proximity to this chromosomal region (9q33) is situated the gene (*NR5A1*) encoding the transcription factor SF-1, a master regulator of adrenocortical and gonadal development [reviewed in Ref. (75, 76)]. Further studies showed that the *NR5A1* gene is amplified and the SF-1 protein is overexpressed in the large majority of Brazilian children ACT (31, 77, 78). Interestingly, the SF-1 protein was overexpressed even in cases lacking gene amplification (31, 78), suggesting that mechanisms in addition to copy number gain may also account for SF-1 overexpression. The dosage-dependent effect of SF-1 in boosting adrenocortical cell proliferation was shown by studies in human cell lines and in transgenic mice (32) by regulation of a fairly large set of dosage-dependent target genes far exceeding its classical steroidogenic targets [(79); reviewed in Ref. (80)]. In children ACT, SF-1 overexpression appears to be a widespread finding, with no relationship with malignancy [(31, 78); see Figure 1]. Conversely, SF-1 overexpression in adult ACT is less common than in children (78) and is an unfavorable prognostic marker (81, 82) (Table 1). Remarkably, SF-1 transcriptional regulatory activity can be pharmacologically targeted leading to a decrease of adrenocortical cancer cell proliferation (83), suggesting that this transcription factor may represent a novel therapeutic target in ACT.

A subsequent SNP array study on both Brazilian and non-Brazilian ACT cases precisely defined recurrent genomic alterations in children ACT (46), the most frequent being loss of 4q34, gain of 9q33-q34 and 19p, and LOH of the whole chromosome 17 (harboring *TP53*) and 11p15 (harboring *IGF2*). Remarkably, a number of focal deletions were detected at 4q34, defining a common deleted region surrounding the non-coding RNA *LINC00290* gene. It is also noteworthy that the extent of the peak region of gain in 9q33-q34 suggests that other genes lying in a telomeric position with respect to *NR5A1* may also be important for ACT pathogenesis. In addition, focal amplifications and homozygous deletions comprising well-known oncogenes (*MYC, MDM2, PDGFRA, KIT, MCL1, BCL2L1*) and tumor suppressors (*TP53, RB1, RPH3AL*) were identified. Although genomic profiles in non-Brazilian tumors with a mutated *TP53* (other than R337H) were similar to Brazilian tumors, those with a wild-type *TP53* displayed distinct genomic alterations, harboring significantly fewer rearrangements. Remarkably, 50% of *TP53* wild-type tumors investigated in this study displayed as sole rearrangement a copy-neutral LOH of the imprinted region at 11p15, providing further evidence for a major role of this region in ACT development.

The landscape of genomic alterations in a worldwide series of children ACT enrolled at IPACTR (84) has been more precisely defined by a very recent study integrating whole exome, whole genome, and RNA-sequencing data (85). This work confirmed LOH in the 11p15 region in the large majority of cases and systematic overexpression of *IGF2*, together with frequent *TP53* mutations, widespread 9q copy number gain, and 4q34 loss. By comparing the mutant allele fraction of SNV in copy-neutral LOH regions to allelic imbalance values, it was possible to establish that in most cases copy-neutral LOH of chromosomes 11p and 17 occurred early during tumorigenesis, suggesting that those events drive tumor formation. Additional recurrent genetic alterations in children ACT were somatic mutations in the *ATRX* (a DNA helicase) and *CTNNB1* genes. Intriguingly, some tumors bore integration of human herpesvirus-6 (HHV6) in the telomeric region of chromosome 11p. A poor outcome was predicted by concomitant *TP53*/*ATRX* mutations and associated genomic abnormalities, including massive structural variations and a high background mutation rate (Table 1).

**Figure 2:**
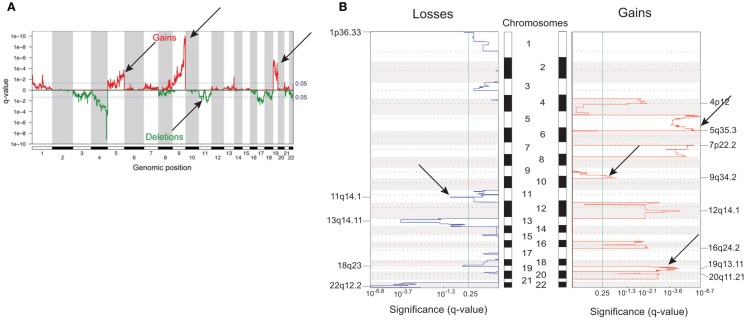
**Differences and similarities in genomic profiles between children and adult ACT**. **(A)** Genomic alterations in children ACT. **(B)** Genomic alterations in adult ACC. Common regions of gains (chr. 5, 9q, 19) and losses (chr. 11) are indicated with arrows. Adapted from Ref. (46, 91) with permission from Endocrine Society Press.

In adult ACT, earlier CGH studies showed a significantly increased prevalence of genomic imbalances in carcinomas compared to adenomas and sometimes contrasting patterns of gain and losses (73, 74, 86–88). CGH array studies evidenced a set of chromosomal aberrations in ACC associated with survival in a fashion dependent on their accumulation (89). Carcinomas were confirmed to harbor a higher number of chromosomal alterations than adenomas (90–92). Recently, activating mutations of the PKA catalytic subunit were shown to be associated with cortisol-secreting adrenocortical adenomas in adults (93–96). In general, gains had a higher impact than losses on gene expression profiles (91). A comparison between genome alterations in children ACT and adult ACC is shown in Figure 2. Methylome studies were also performed in adult ACC (97–99). According to their DNA methylation levels, malignant tumors could be divided into two groups, one displaying low and the other one elevated levels of methylation in CpG islands (CpG island methylator phenotype, CIMP). This hypermethylated tumors group could in turn be subdivided into two subgroups (CIMP-high and CIMP-low), which had prognostic relevance, with the CIMP-high phenotype clearly being associated to worse prognosis (99).

A study integrating transcriptome, miRNome, copy number alterations, methylome, and whole exome sequencing data in adult ACC was recently published (66), showing that major pathways involved by mutation or homozygous deletion include beta-catenin (*CTNNB1* and *ZNRF3*), p53/Rb signaling (*TP53*, *CDKN2A*, and *RB1*), and chromatin remodeling (*MEN1*, *DAXX*, *ATRX*, *MED12*, and *TERT*) (Table 1). In addition, recurrent homozygous deletions were found in 4q34, similarly to children ACT. This study also showed that a substantial overlap exists among the different omics classifications of ACC: the previously identified gene expression profile clusters (C1A, C1B, and C2; see section on *Distinct Patterns of Coding Genes Expression in Children vs. Adult ACT*) (54) are strongly correlated with subgroups based on DNA methylation and miRNA expression, mutation rate, and alteration of key molecular pathways.

## Perspectives

Children ACT represent a distinct pathological entity compared to tumors in adults concerning their origin, clinical manifestations, molecular alterations, and prognostic evolution. Important fields of investigation in the future will be the search for genetic and environmental factors that modulate penetrance of ACT in carriers of germline *TP53* mutations in order to orient screening procedures to detect disease at an early stage, the identification of robust biomarkers of malignancy, which are still lacking, and the clinical testing of targeted therapies against the major molecular pathways that are altered in this disease (100).

## Conflict of Interest Statement

The authors declare that the research was conducted in the absence of any commercial or financial relationships that could be construed as a potential conflict of interest.
